# Feasibility of transoral robotic selective neck dissection with or without a postauricular incision for papillary thyroid carcinoma: A pilot study

**DOI:** 10.3389/fsurg.2022.985097

**Published:** 2022-10-11

**Authors:** Kyung Tae, Hae Won Choi, Yong Bae Ji, Chang Myeon Song, Jung Hwan Park, Dong Sun Kim

**Affiliations:** ^1^ Department of Otolaryngology-Head and Neck Surgery, College of Medicine, Hanyang University, Seoul, South Korea; ^2^ Department of Internal Medicine, College of Medicine, Hanyang University, Seoul, South Korea

**Keywords:** robotic thyroidectomy, selective neck dissection, papillary thyroid carcinoma, thyroid cancer, transoral thyroidectomy

## Abstract

**Background:**

The study aimed to evaluate the feasibility of transoral robotic selective neck dissection (SND) with or without a postauricular incision for papillary thyroid carcinoma (PTC).

**Methods:**

We studied 14 patients with PTC who underwent robotic SND *via* the transoral or combined transoral and postauricular approaches.

**Results:**

The transoral approach was performed on 10 patients for dissection of levels III and IV. An additional postauricular incision was made on 4 patients for dissection of level II in addition to levels III, IV, and V. The operation was completed successfully in 13 patients, except 1 patient with the procedure conversion due to uncontrolled bleeding from the internal jugular vein. The mean numbers of removed lymph nodes in the lateral compartment were 23.1 ± 9.4 and 38.3 ± 8.5 in the transoral and combined groups. Transient recurrent laryngeal nerve palsy occurred in 1 patient, transient hypoparathyroidism in 3 patients, and chyle leakage in 1 patient. There were no hematomas, mental nerve injuries, surgical space infections, or CO_2_ embolisms.

**Conclusion:**

Transoral robotic SND is feasible with or without a postauricular incision.

## Introduction

Advances in minimally invasive head and neck surgery and increased concern for postoperative cosmesis have led to the development of endoscopic and robotic surgery for thyroid tumor treatment during the past 20 years (1, 2). In particular, transoral robotic and endoscopic thyroidectomies have gained popularity worldwide (1, 3, 4). The surgical morbidity related to creating the working space is less invasive with the transoral approach than other remote-access approaches (4, 5). Significant advantages of transoral thyroidectomy compared with conventional thyroidectomy include superior cosmesis and possibility of better postoperative voice outcomes (6–8).

Traditionally, lateral neck dissection for thyroid cancer was usually performed using a hockey stick incision or single transverse incision (9). These incisions provide an excellent surgical view for comprehensive lateral neck dissection. However, the conventional transcervical approach leaves a neck scar, and furthermore, some scars heal with hypertrophy. On the extension of remote access thyroidectomy, various remote access approaches have been developed for lateral neck dissection to avoid visible long neck scarring. In the history of robotic and endoscopic lateral neck dissection for thyroid cancer, a minimally invasive video-assisted lateral neck dissection approach was reported in 2007 (10). Since then, robot-assisted lateral neck dissection *via* gasless axillary, breast, anterior chest, and retroauricular approaches have been developed (11, 12). However, lateral neck dissection *via* the transoral approach has been considered difficult. After becoming familiar with the transoral thyroidectomy procedure, we expanded the transoral robotic approach to lateral neck dissection for papillary thyroid carcinoma (PTC) (13).

This study aimed to analyze and evaluate the feasibility and early surgical outcomes of transoral robotic selective neck dissection (SND) with or without a postauricular incision for PTC.

## Materials and methods

This study included 14 patients who underwent transoral robotic SND with or without a postauricular incision using the da Vinci Si surgical system (Intuitive Surgical, Inc., Sunnyvale, CA, USA) from February 2019 through September 2021. All patients were confirmed to have PTC on the final pathology report. Of 14 patients, 10 performed a transoral robotic approach for SND of levels III and IV (with/without level V). Four patients underwent SND of levels II, III, and IV (with/without level V) by the combined transoral and postauricular approach. The decision to perform transoral robotic SND was based on the extent of disease, patient preferences, and financial reasons. Each patient gave informed consent for the possibility of conversion to conventional transcervical or other types of remote-access thyroidectomy and neck dissection. This study was approved by the institutional review board of Hanyang University Hospital.

The indications for transoral robotic SND included PTC patients with metastatic lymph nodes in level III or IV, confirmed by fine-needle aspiration cytology. If there were metastatic lymph nodes at level II, an additional postauricular incision was made to perform level-II dissection, in addition to dissection of levels III, IV, and V. Exclusion criteria of transoral robotic SND included large conglomerated metastatic lymph nodes in the lateral compartment, metastatic lymph nodes with extensive invasion of surrounding structures or extensive multi-level involvement, recurrent tumors, distant metastases, and a history of neck irradiation or surgery.

We evaluated patient demographics, tumor characteristics, the extent of surgery, the number of removed lymph nodes and positive nodes, operative time, conversion of the procedure, complications, radioactive iodine (RAI) ablation status, and recurrence status.

Vocal fold mobility was elevated using flexible laryngoscopy preoperatively and postoperatively in all patients. Hypoparathyroidism was defined as any drop in the blood parathyroid hormone level below the normal limit regardless of hypocalcemia symptoms. Permanent recurrent laryngeal nerve (RLN) palsy and hypoparathyroidism was defined if it did not resolve within 6 months. Seroma was defined as when the total amount of aspirated fluid was over 20 ml.

### Operative procedures of transoral robotic SND

**Figure 1:**
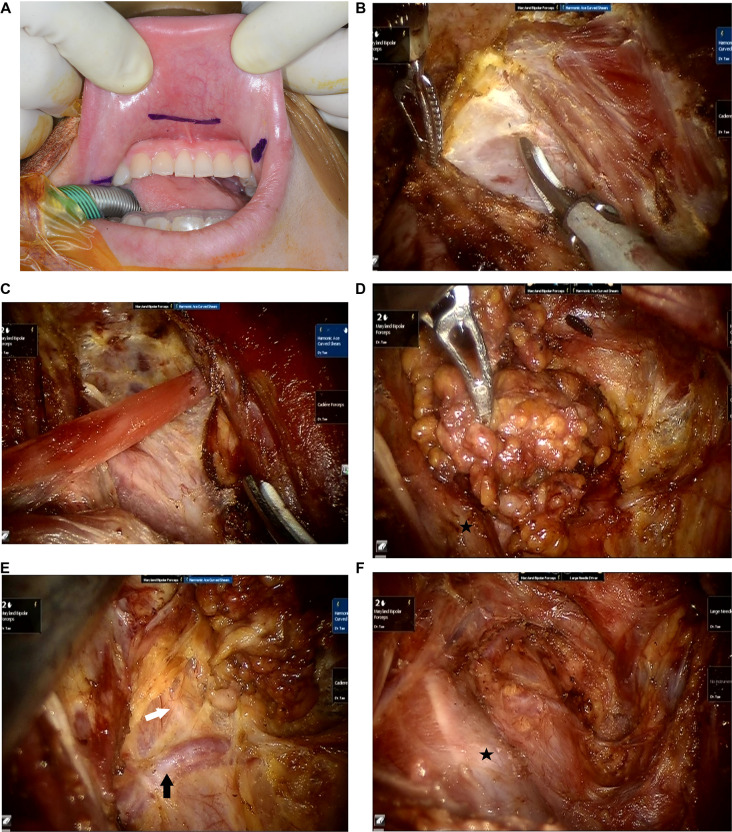
Operative procedure of transoral robotic selective neck dissection. (**A**) Three vestibular incisions are made. (**B**) The fascia overlying the sternocleidomastoid muscle is dissected to expose the internal jugular vein. (**C**) The omohyoid muscle is exposed and cut. (**D**) Lymph nodes and fibrofatty tissue of levels III and IV are dissected. (**E**) The transverse cervical artery and the phrenic nerve are preserved. Black arrow: transverse cervical artery, white arrow: phrenic nerve. (**F**) Surgical view after completion of selective neck dissection of levels III and IV. Black star: internal jugular vein.

Transoral robotic SND was described in our previous report (13). Briefly, the patient is placed in a supine position with neck extension. General anesthesia is induced with orotracheal intubation. Standardized intermittent intraoperative neural monitoring is performed on all patients. A 1.5-cm central incision is made 1 cm above the base of the lower lip frenulum, and 2 lateral incisions are made close to the oral commissure to avoid injuring the mental nerve (Figure 1A). After inserting 3 trocars *via* vestibular incisions, the exact plane of the subplatysmal layer is identified in the submental area, and the skin flap is elevated through the plane of the subplatysmal layer under an endoscopic procedure. After elevating the skin flap in the submental area, a da Vinci Si surgical system (Intuitive Surgical, Inc., Sunnyvale, CA, USA) is docked; a 30-degree endoscope is placed in the center, and 2 robotic instruments are placed on either side of the endoscope. The elevation of the skin flap is continued inferiorly to the level of the sternal notch and clavicle and laterally to the posterior border of the sternocleidomastoid (SCM) muscle. After creating sufficient working space, a long robotic trocar is inserted through a 1-cm incision made in the right axillary fossa to place a third robotic instrument.

After completion of thyroidectomy and CND as usual manner, lateral SND is performed. The fascia between the strap muscles and SCM muscle is dissected to expose levels III and IV (Figure 1B). An external hanging suture is applied to retract the SCM muscle laterally to obtain a better surgical view, if necessary. The omohyoid muscle is cut (Figure 1C), and the lymph nodes and fibrofatty tissues in levels III and IV are dissected. (Figure 1D).

The cervical plexus nerves are preserved if possible. The transverse cervical artery is preserved, and the phrenic nerve, which passes under the deep cervical fascia, is preserved (Figure 1E). Additionally, lymph nodes of level VB are dissected if there are suspicious lymph nodes. After completing SND including in levels III and IV (Figure 1F), the resected specimen is extracted in a plastic bag *via* the axillary port. A drain is placed through the axillary incision. Finally, the oral vestibular incisions and the axillary incision are closed.

### Operative procedures of transoral robotic SND with a gasless postauricular approach

**Figure 2:**
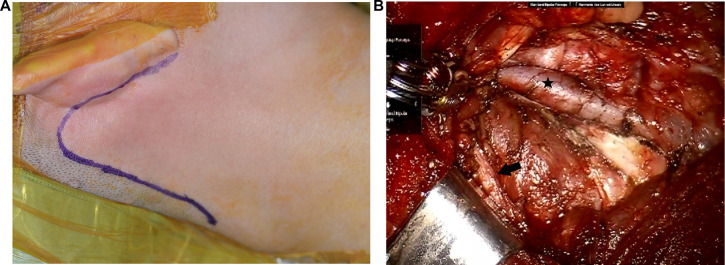
Transoral robotic selective neck dissection with a postauricular incision. (**A**) An additional postauricular incision is made. (**B**) Surgical view after dissection of level II. Black star: internal jugular vein, black arrow: spinal accessory nerve.

For patients with metastatic lymph nodes in level II, the gasless postauricular approach is added to the transoral approach to dissect level II lymph nodes. Thyroidectomy, CND, and dissection of levels III, IV, and V are performed using the transoral approach with CO_2_ insufflation as described above. For the gasless postauricular approach, the da Vinci robot is de-docked, and the patient’s head is turned to the opposite side of the lesion. A postauricular incision is made in the postauricular sulcus and extends to the occipital hairline (Figure 2A). The skin flap is elevated at the postauricular area and over the SCM muscle by monopolar cautery under direct vision. Elevation of the skin flap is connected to the surgical space created previously during the transoral approach. Then, an external retractor (Meditech Inframed, Seoul, Korea) is inserted underneath the skin flap to maintain working space without CO_2_ insufflation. The da Vinci robot is docked again. Three robotic arms are placed through the postauricular incision, including a 30-degree endoscope in the center and 2 robotic instruments on either side of the endoscope. The level-IIA lymph nodes are dissected, and the spinal accessory nerve is identified and preserved at level II (Figure 2B). The level-IIB lymph nodes are also dissected if indicated with adequate retraction of the SCM muscle by a surgical assistant.

## Results

### Clinicopathological characteristics

The clinicopathological characteristics of patients and tumors are listed in Table 1. There were 11 women and 3 men. The mean age was 37.3 ± 11.9 years, and the mean body mass index was 23.9 ± 3.9. The mean diameter of the largest primary tumor was 12.6 ± 11.6 mm. Multifocal primary tumors were found in 57.1% of patients and bilateral tumors in 28.6% of patients. Minimal ETE was found in 64.3% of patients, and maximal ETE was not encountered. Eleven (78.6%), 1 (7.1%), and 2 (14.3%) patients were staged as T1, T2, and T3, respectively. The postoperative N classification was N1b in 14 patients. All patients were stage I.

**Table 1:** Clinicopathological characteristics of the patients with papillary thyroid carcinoma.

Characteristics	Number of patients (%) (*N* = 14)
Age (years)	37.3 ± 11.9
**Sex**	
Male	3 (21.4%)
Female	11 (78.6%)
Body mass index (kg/m^2^)	23.9 ± 3.9
**Pathology**	
Papillary carcinoma	14 (100%)
Size of tumor (mm)	12.6 ± 11.6
Tumor multiplicity	8 (57.1%)
Tumor bilaterality	4 (28.6%)
Lymphovascular invasion	6 (42.9%)
Minimal extrathyroidal extension	9 (64.3%)
**T classification**	
T1	11 (78.6%)
T2	1 (7.1%)
T3	2 (14.3%)
T4	0 (0%)
**N classification**	
N0	0
N1b	14 (100%)
**Stage**	
Stage I	14 (100%)
Stage II	0
Stage III	0
Stage IV	0

### Surgical outcomes

The surgical outcomes are summarized in Table 2. The operation was completed successfully in 13 patients, except 1 patient. In 1 patient who underwent the transoral approach, the operation was converted to the gasless postauricular approach due to uncontrollable bleeding from the internal jugular vein.

**Table 2:** Surgical outcomes of patients with papillary thyroid carcinoma who underwent transoral robotic selective neck dissection.

Cases	1	2	3	4	5	6	7	8	9	10	11	12	13	14
Sex/age	F/23	F/53	F/42	M/43	F/35	F/42	F/28	F/48	F/55	F/22	M/30	M/53	F/25	F/23
Surgical approach	TO	TO and conversion to PA	TO	TO	TO	TO	TO	TO	TO	TO	TO + PA	TO + PA	TO + PA	TO + PA
Extent of thyroidectomy	TT	TT	TT	TT	Lob	TT	TT	TT	TT	TT	TT	TT	TT	TT
Extent of CND	Bilat	Unilat	Unilat	Bilat	Unilat	Unilat	Bilat	Bilat	Bilat	Bilat	Bilat	Bilat	Bilat	Unilat
No. of removed LN in CND	7	3	9	17	6	5	12	21	6	11	12	14	11	10
No. of positive LN in CND	2	2	1	16	0	3	6	11	0	7	6	9	7	5
Extent of SND (levels)	III, IV	III, IV	III, IV	III, IV	III, IV	III, IV, V	III, IV	III, IV	III, IV	III, IV	II, III, IV, V	II, III, IV	II, III, IV, V	II, III, IV
No. of removed LN in SND (levels)	10 (III 4, IV 6)	20 (III 3, IV 17)	20 (III 11, IV 9)	14 (III 6, IV 8)	26 (III 11, IV 15)	35 (III 18, IV 11, V 6)	29 (III 12, IV 17)	40 (III 16, IV 24)	20 (III 9, IV 11)	17 (III 6, IV 11)	44 (II 14, III, 8 IV 16, V 6)	30 (II 9, III 13, IV 8)	47 (II 13, III 15, IV 16, V 3)	32 (II 6, III 18, IV 8)
No. of positive LN in SND (levels)	3 (IV 3)	2 (III 1, IV 1)	3 (IV 3)	3 (III 1, IV 2)	1 (IV 1)	8 (III 3, IV 5)	1 (IV 1)	3 (III 1, IV 2)	2 (IV 2)	3 (III 3)	7 (II 1, III 2, IV 4)	7 (II 1, III 5, IV 1)	12 (II 2, III 5, IV 3, V 2)	7 (II 1, III 5, IV 1)
Operative time (min)	320	610	310	360	275	235	295	330	300	270	390	510	415	410
RAI ablation	Yes	Yes	Yes	Yes	No	Yes	Yes	Yes	Yes	Yes	Yes	Yes	Yes	Yes
Stimulated Tg at RAI ablation (ng/ml)	10	1.06	33.4	18	NA	42	21.1	2.5	0.23	32	30	96	13.7	61
Stimulated Tg at diagnostic WBS after RAI ablation (ng/ml)	3.9	<0.04	2.84	NA	NA	NA	15.05	4.21	<0.04	9.5	<0.04	2.4	2.72	3.8
Complications	None	Conversion to PA, Seroma	None	VCP	None	None	None	Hypopara, Chyle leakage	Hypopara	Seroma	Seroma	None	Seroma	Hypopara

TO, transoral; PA, postauricular; TT, total thyroidectomy; Lob, lobectomy; LN, lymph node; CND, central neck dissection; No, number; SND, selective neck dissection; Bilat, bilateral; Unilat, unilateral; RAI, radioactive iodine; Tg, thyroglobulin; NA, not applicable; WBS, whole body scan; VCP, vocal cord palsy; Hypopara, hypoparathyroidism.

In terms of surgical extent, total thyroidectomy was performed in 13 patients, and lobectomy was performed in 1 patient who strongly preferred preserving the opposite thyroid lobe and avoiding thyroid hormone replacement therapy. All patients underwent concomitant unilateral SND and therapeutic or prophylactic CND. Dissection of levels III and IV was performed on 9 patients, dissection of levels II–IV on 2 patients, levels II–V on 2 patients, and levels III–V on 1 patient. The SND was on the left side in 5 patients and on the right in 9 patients.

Central compartment lymph node metastasis occurred in 12 patients (85.7%). In the solely transoral approach group, the mean numbers of removed and positive lymph nodes in the central compartment were 9.7 ± 5.7 and 6.0 ± 5.2, respectively. The mean numbers of removed and positive lymph nodes in the lateral compartment were 23.1 ± 9.4 and 2.9 ± 1.9, respectively. In the combined transoral and postaurciular approach group, the mean numbers of removed and positive lymph nodes in the central compartment were 11.8 ± 1.7 and 6.8 ± 1.7, respectively. The mean numbers of removed and positive lymph nodes in the lateral compartment were 38.3 ± 8.5 and 8.3 ± 2.5, respectively. In the 14 lateral necks, level-IV metastasis occurred in 13 patients, level-III metastasis in 9 patients, level II metastasis in 4 patients, and level V metastasis in 1 patient.

The mean total operative time was 299 ± 37 min in the transoral group and 431 ± 54 min in the combined group. As for postoperative complications, transient RLN palsy occurred in 3.7% (1/27 nerves at risk), and transient hypoparathyroidism occurred in 3 patients (21.5%). No patients had permanent RLN palsy or hypoparathyroidism. Postoperative chyle leakage occurred in 1 patient, and it was controlled by conservative treatment. Postoperative seroma formation occurred in 4 patients (28.6%), all of whom were treated by repeated aspiration until their seromas resolved. There were no hematomas, mental nerve injuries, surgical space infections, or CO_2_ embolisms. Postoperative RAI ablation was performed on 13 patients (92.8%), except 1 patient who underwent thyroid lobectomy. The mean stimulated thyroglobulin (Tg) level at RAI ablation was 27.8 ± 26.3 ng/ml, and the mean thyroid stimulating hormone (TSH) level was 99.8 ± 64.8 μIU/ml. The mean stimulated Tg at diagnostic whole body iodine scan after RAI ablation was 4.05 ± 4.77 ng/ml, and the mean TSH level was 109.2 ± 59.7 μIU/ml. After a mean follow-up of 14.5 months, there were no structural recurrences on imaging studies.

## Discussion

We developed a novel transoral robotic approach for SND after familiarizing ourselves with the transoral approach for thyroidectomy and CND (13). As shown in this study, transoral robotic SND of levels III and IV is feasible, and dissection of level V is also possible if indicated. However, dissection of level II lymph nodes is challenging due to the inadequate axis of the surgical view and instruments. Therefore, we added a postauricular approach without CO_2_ insufflation to the transoral approach to reach level II lymph nodes. The additional gasless postauricular approach was useful and practical for performing level II dissection.

The optimal extent of therapeutic SND for PTC has not been clearly established, although most surgeons agree that berry-picking should be avoided. The American Thyroid Association (ATA) guidelines recommend therapeutic lateral neck dissection for patients with biopsy-proven metastasis to the lateral compartment lymph nodes (14). However, the ATA guidelines do not provide any recommendations regarding the extent of lateral SND. Generally, level-I dissection is not recommended because metastasis to level I is rare. Metastases to the lateral compartment usually occur at levels II–V in PTC, most commonly at levels III and IV (14, 15). Therefore, many surgeons recommended comprehensive SND, including levels II–V or at least levels IIA, III, and IV, for the complete clearance of lateral compartment metastases (15, 16). The merits of routine dissection of levels IIB and V are debated because of the relatively low metastatic rates and potential morbidity associated with injury to the spinal accessory nerve.

Super-SND or single-level SND of levels III and IV is also suggested for PTC patients with single-level metastatic lymph nodes in level III or IV. This concept is based on minimal surgical morbidity without compromising the oncologic outcomes and the relatively low metastatic rates of levels II or V (17–19). One study conducted by Kim et al. analyzed 241 PTC patients with lateral compartment lymph node metastasis and demonstrated that solitary lateral lymph node metastasis occurred in 20.7% of patients (17). Related factors to solitary lateral lymph node metastases were metastatic lymph node size less than 0.7 cm and a lack of ETE (17). Consequently, the authors suggested that single-level SND can be an alternative to comprehensive lateral neck dissection in such patients. Another study also analyzed the solitary lateral compartment metastasis rate and characteristics in 391 PTC patients (18). In their study, 11.3% of patients had solitary lateral lymph node metastasis, which was associated with age ≥47 years, a lack of capsular invasion, and an absence of central lymph node metastasis (18).

Indeed, we support comprehensive lateral SND, including levels II–V, for PTC patients with palpable lateral lymph node metastasis or multi-level metastasis in the lateral compartment. However, we also support single-level or super-SND of levels III and IV for solitary or single-level small lymph node metastasis at level III or IV. Therefore, transoral robotic SND can be indicated for patients with single-level lymph node metastasis in level III or IV, based on previous studies (13, 17–19).

Two articles, including a cohort study with 20 patients conducted by Tan et al. and another case report performed by Ngo et al., reported transoral endoscopic SND for dissection of levels III and IV (20, 21). However, the robotic procedure is more effective for performing SND than the endoscopic procedure. The number of removed lateral compartment lymph nodes in our transoral robotic procedure seems to be higher compared to that of the transoral endoscopic SND procedure (23.1 ± 10.6 in our robotic procedure and 10.9 ± 2.8 in the endoscopic procedure by Tan et al.) (20). The da Vinci robot provides superior high-density 3-dimensional visualization and magnification and 7-degree freedom of the instrument movement with tremor filtration (1, 22). Also, it enables 3-hand surgery with the placement of a third robotic instrument for counter traction. Therefore, the robotic procedure enhances surgical dexterity and facilitates tissue dissection. Significantly, the robotic procedure has advantages in creating the working space and dissecting the upper pole area. Consequently, we strongly prefer the robotic procedure rather than the endoscopic procedure (22). However, the operative time of our transoral robotic SND seems to be longer than that of conventional transcervical or transoral endoscopic SND (299 ± 37 min in this study and 146 ± 19 min in the endoscopic procedure) (20). The longer operative time of the robotic procedure might be related to the docking time of the surgical robot and the relatively long time needed to exchange the robotic instrument and endoscopy.

In the transoral SND, dissection of level II lymph nodes is challenging due to the inadequate axis of the surgical view and instruments. One case report presenting the surgical video of transoral endoscopic SND suggested even level-II dissection is possible *via* the transoral endoscopic approach, in addition to dissection of levels III and IV (21). Indeed, level-II dissection might be possible solely *via* the transoral approach in selected patients. However, we could not confirm that level II dissection can be performed completely in every patient. This study showed that the addition of the gasless postauricular approach to the transoral approach is effective and practical for the complete dissection of level II. Someone might argue that the postauricular approach is enough in performing SND and total thyroidectomy rather than the combined transoral and postauricular approach. However, the combined approach has more advantages than the postauricular approach. The combined approach is more effective in performing total thyroidectomy than the solely postauricular approach because it is challenging to perform contralateral total lobectomy *via* a unilateral postauricular incision. In addition, additional skin flap elevation *via* a postauricular incision is faster than the standard gasless postauricular approach for comprehensive lateral neck dissection. It is limited to the postauricular and level-II area in the combined approach.

Generally, the technique of transoral robotic SND is challenging compared to the conventional approach due to the limited surgical space and manipulation of the instruments. In this study, there was 1 case of conversion during the transoral robotic SND procedure due to uncontrollable bleeding from minor tearing of the internal jugular vein. The internal jugular vein was torn during dissection of the level-IV area, and it was impossible to stop the bleeding. Therefore, we converted the procedure to the gasless postauricular approach. We decided to convert the procedure to the postauricular approach rather than the conventional approach because, during preoperative counseling, the patient strongly expressed a preference for scarless neck surgery. While compressing the level-IV area to minimize bleeding from the internal jugular vein, a postauricular incision was made, and the skin flap was elevated. The bleeding point of the internal jugular vein was sutured to control bleeding under direct vision. Surgeons need to keep in mind the possibility of conversion during the transoral SND procedure due to uncontrollable intraoperative bleeding or the inability to eradicate metastatic lymph nodes.

At the time of writing, there were no recurrences after robotic SND, although the follow-up period was short (mean, 14.5 months). At the RAI ablation, the mean stimulated Tg level was 27.8 ± 26.3 ng/ml (ranges, 0.23–96). The mean stimulated Tg at diagnostic whole body iodine scan after RAI ablation was 4.05 ± 4.77 ng/ml (ranges, <0.04–15.1). Further studies are necessary to evaluate the surgical completeness and oncologic safety of this transoral robotic SND procedure, comparing the stimulated Tg level, recurrence, and survival with the conventional approach.

This study has some limitations. First, this was a pilot study to evaluate the feasibility and early surgical outcomes. The sample size was too small to reach a firm conclusion. Second, this is not a comparative study with the control group. Therefore, further larger-scale comparative studies with long-term follow-up are necessary to determine the surgical and oncologic outcomes. However, despite limitations to this study, it might be significant that this study demonstrated the feasibility of transoral SND with or without a postauricular incision using the surgical robot in PTC for the first time, based on early surgical outcomes.

## Conclusion

Transoral robotic SND is feasible for dissection of levels III and IV. Adding the postauricular approach is effective for dissecting level II. Further large-scale studies with long-term follow-up should be necessary to clarify the results of this study and determine the long-term surgical and oncologic outcomes.

## Data Availability

The original contributions presented in the study are included in the article/Supplementary Material, further inquiries can be directed to the corresponding author/s.

## References

[1] Tae K Ji YB Song CM Ryu J. Robotic and endoscopic thyroid surgery: evolution and advances. Clin Exp Otorhinolaryngol. (2019) 12:1–11. 10.21053/ceo.2018.00766 30196688 PMC6315214

[2] Tae K. Robotic thyroid surgery. Auris Nasus Larynx. (2021) 48:331–8. 10.1016/j.anl.2020.06.007 32636045

[3] Anuwong A Ketwong K Jitpratoom P Sasanakietkul T Duh QY. Safety and outcomes of the transoral endoscopic thyroidectomy vestibular approach. JAMA Surg. (2018) 153:21–7. 10.1001/jamasurg.2017.3366 28877292 PMC5833624

[4] Tae K. Transoral thyroidectomy: is it a real game changer? Clin Exp Otorhinolaryngol. (2020) 13:93–4. 10.21053/ceo.2020.00402 32434309 PMC7248618

[5] Tae K Ji YB Song CM Park JS Park JH Kim DS. Safety and efficacy of transoral robotic and endoscopic thyroidectomy: the first 100 cases. Head Neck. (2020) 42:321–9. 10.1002/hed.25999 31682312

[6] Lee DW Bang HS Jeong JH Kwak SG Choi YY Tae K. Cosmetic outcomes after transoral robotic thyroidectomy: comparison with transaxillary, postauricular, and conventional approaches. Oral Oncol. (2021) 114:105139. 10.1016/j.oraloncology.2020.105139 33460884

[7] Song CM Park JS Park HJ Tae K. Voice outcomes of transoral robotic thyroidectomy: comparison with conventional trans-cervical thyroidectomy. Oral Oncol. (2020) 107:104748. 10.1016/j.oraloncology.2020.104748 32371263

[8] Tae K. Complications of transoral thyroidectomy: overview and update. Clin Exp Otorhinolaryngol. (2021) 14:169–78. 10.21053/ceo.2020.02110 33211953 PMC8111399

[9] Song CM Ji YB Kim IS Lee JY Kim DS Tae K. Low transverse incision for lateral neck dissection in patients with papillary thyroid cancer: improved cosmesis. World J Surg Oncol. (2017) 15:97. 10.1186/s12957-017-1160-1 28472951 PMC5418722

[10] Lombardi CP Raffaelli M Princi P De Crea C Bellantone R. Minimally invasive video-assisted functional lateral neck dissection for metastatic papillary thyroid carcinoma. Am J Surg. (2007) 193:114–8. 10.1016/j.amjsurg.2006.02.024 17188101

[11] Zhang Z Sun B Ouyang H Cong R Xia F Li X. Endoscopic lateral neck dissection: a new frontier in endoscopic thyroid surgery. Front Endocrinol. (2021) 12:796984. 10.3389/fendo.2021.796984 PMC8728058 35002974

[12] Song CM Ji YB Sung ES Kim DS Koo HR Tae K. Comparison of robotic versus conventional selective neck dissection and total thyroidectomy for papillary thyroid carcinoma. Otolaryngol Head Neck Surg. (2016) 154:1005–13. 10.1177/0194599816638084 26980906

[13] Tae K Kim KH. Transoral robotic selective neck dissection for papillary thyroid carcinoma: dissection of levels III and IV. Head Neck. (2020) 42:3084–8. 10.1002/hed.26379 32794247

[14] Haugen BR Alexander EK Bible KC Doherty GM Mandel SJ Nikiforov YE 2015 American thyroid association management guidelines for adult patients with thyroid nodules and differentiated thyroid cancer: the American thyroid association guidelines task force on thyroid nodules and differentiated thyroid cancer. Thyroid. (2016) 26:1–133. 10.1089/thy.2015.0020 26462967 PMC4739132

[15] Keum HS Ji YB Kim JM Jeong JH Choi WH Ahn YH Optimal surgical extent of lateral and central neck dissection for papillary thyroid carcinoma located in one lobe with clinical lateral lymph node metastasis. World J Surg Oncol. (2012) 10:221. 10.1186/1477-7819-10-221 23098385 PMC3544686

[16] Javid M Graham E Malinowski J Quinn CE Carling T Udelsman R Dissection of levels II through V is required for optimal outcomes in patients with lateral neck lymph node metastasis from papillary thyroid carcinoma. J Am Coll Surg. (2016) 222:1066–73. 10.1016/j.jamcollsurg.2016.02.006 27049777

[17] Kim SM Chun KW Chang HJ Kim BW Lee YS Chang HS Solitary lateral neck node metastasis in papillary thyroid carcinoma. World J Surg Oncol. (2014) 12:109. 10.1186/1477-7819-12-109 24755464 PMC4016639

[18] Yang Q Chen P Hu HY Tan HL Li GY Liu M Preoperative sonographic and clinicopathological predictors for solitary lateral neck node metastasis in papillary thyroid carcinoma: a retrospective study. Cancer Manag Res. (2020) 12:1855–62. 10.2147/CMAR.S244406 32210628 PMC7075331

[19] Kim H Jin YJ Cha W Ahn SH. Feasibility of super-selective neck dissection for indeterminate lateral neck nodes in papillary thyroid carcinoma. Head Neck. (2014) 36:487–91. 10.1002/hed.23320 23729272

[20] Tan Y Guo B Deng X Ding Z Wu B Niu Y Transoral endoscopic selective lateral neck dissection for papillary thyroid carcinoma: a pilot study. Surg Endosc. (2020) 34:5274–82. 10.1007/s00464-019-07314-8 31834511

[21] Ngo DQ Tran TD Le DT Ngo QX Van Le Q. Transoral endoscopic modified radical neck dissection for papillary thyroid carcinoma. Ann Surg Oncol. (2021) 28:2766. 10.1245/s10434-020-09466-7 33462715

[22] Tae K Lee DW Song CM Ji YB Park JH Kim DS Early experience of transoral thyroidectomy: comparison of robotic and endoscopic procedures. Head Neck. (2019) 41:730–8. 10.1002/hed.25426 30521674

